# Fabrication of Sputtered Ce/La, La/Ce Oxide Bilayers on AA6061 and AA7075 Aluminum Alloys for the Development of Corrosion Protective Coatings

**DOI:** 10.3390/ma11071114

**Published:** 2018-06-29

**Authors:** Silvia B. Brachetti-Sibaja, Miguel A. Domínguez-Crespo, Aidé M. Torres-Huerta, Sandra E. Rodil-Posada, Ana B. López-Oyama, David S. García-Zaleta, Edgar Onofre-Bustamante

**Affiliations:** 1 Instituto Politécnico Nacional, Centro de Investigación en Ciencia Aplicada y Tecnología Avanzada, Unidad Altamira. km 14.5 Carr. Tampico-Puerto Industrial Altamira, Altamira 89600, Mexico; silvia.brachetti@itcm.edu.mx (S.B.B.-S.); atorresh@ipn.mx (A.M.T.-H.); eonofre@ipn.mx (E.O.-B.); 2 TecNM, Instituto Tecnológico de Ciudad Madero, Ave. Primero de Mayo s/n, Col. Los Mangos 89440, Mexico; 3 UNAM, Instituto de Investigaciones en Materiales, Circuito Exterior s/n C.U., Coyoacán 04510, Mexico; srodil@unam.mx; 4 Conacyt-Instituto Politécnico Nacional, Centro de Investigación en Ciencia Aplicada y Tecnología Avanzada, Unidad Altamira km 14.5 Carr. Tampico-Puerto, Industrial Altamira, Altamira 89600, Mexico; ablopezoy@conacyt.com; 5 Universidad Juárez Autónoma de Tabasco, División Académica Multidisciplinaria de Jalpa de Méndez, Carr. Estatal Libre VHS-COM. km. 27+000 s/n Ranch. Ribera Alta, Jalpa de Méndez, Tabasco 86205, Mexico; david.garcia@ujat.mx

**Keywords:** magnetron sputtering, rare earth, corrosion, thin film, bilayer, CeO_2_, La_2_O_3_, cerium oxide, lanthanum oxide

## Abstract

This work provides a comparative study on the corrosion protection efficiency of Ce, La films as well as Ce/La and La/Ce oxide bilayered coatings deposited onto AA7075 and AA6061 substrates by the radio frequency (RF) magnetron sputtering technique. The coating thickness ranged approximately from 12 to 835 nm, which changed with the deposition parameters and substrate composition. The relationship between microstructure, roughness and electrochemical performance is examined. The reactivity and crystallinity of rare earth (RE) films can be tailored by adjusting the sputtering parameters. Sputtered La films with thickness ca. 390 nm and average roughness of 66 nm showed the best corrosion protection properties in chloride medium as determined by potentiodynamic curves and electrochemical impedance spectroscopy (EIS). The method to obtain RE bilayered coatings, i.e., La/Ce or Ce/La as well as the substrate composition and applied power conditioned their inhibition properties. The RE bilayered coatings displayed better barrier properties than Ce films, which were poorer than those featured by La films.

## 1. Introduction

Rare earth (RE)-based technology has been extensively investigated as a replacement of chromate conversion coatings for both corrosion protection and pretreatments prior to painting different metallic substrates [1,2,3,4,5]. Among them, aluminum alloys for aircraft industry require effective environmentally friendly inhibitors to prevent pitting corrosion [6]. Cerium and lanthanum salts are the most common coatings used to inhibit the corrosion on aluminum alloys.

The corrosion pathway of these lanthanides has also been widely discussed and it is associated with the cathodic areas blocked by precipitation of a film of lanthanide oxide/hydroxide [7,8,9,10], but it strongly depends on the metallic substrate. Additionally, a variety of approaches enables the deposition of metallic coatings containing rare earth (RE) elements for corrosion protection [11,12,13,14,15,16,17,18,19,20,21,22,23,24,25]. However, some limitations of RE coatings obtained by conventional methods are: (i) the precipitation of an insoluble protective RE oxide/hydroxide layer that produces coatings with irregular characteristics; and (ii) the presence of cracks that can penetrate the entire cross-section of the layer. These cracks represent preferential pathways to attack the substrate by aggressive corrosive species [26]. Radio frequency (RF) magnetron sputtering is one of the most used physical approaches used to obtain thin films and could be favorable for fabricating coatings on large scale because of their uniformity, easy fabrication, adhesion, and control parameters [27]. Changes in growth, morphology, and/or chemical composition of the films to produce valuable properties can also be modulated using RF magnetron sputtering [28,29,30].

Undoubtedly, with the rapid development of the aerospace industry, reducing weights, increasing reliability and stability, a proper option of structured materials have become more vital for the success of advanced designs. In this way, considering that RF magnetron sputtering is a scarcely explored technique for inhibiting the corrosion of metallic substrates [26,31,32,33,34,35], it can be an alternative to search new methods to protect metallic substrates for industrial applications. The present work was aimed at using the RF magnetron sputtering technique to evaluate the inhibition efficiency of Ce, La films and bilayered coatings (Ce/La and La/Ce) in order to delay the pitting corrosion of AA7075 and AA6061 aluminum alloys. Due to the microstructure (including chemical composition) of REs coatings, it is fundamental to understand the role played by REs as corrosion inhibitors (CIs) and thus prepare effective RE-containing inhibitors once the dependence of microstructural and corrosion properties has been investigated. To characterize properly the structure, morphology, topography, and chemical composition of the sputtered coatings, samples were also deposited on glass and silicon (100) substrates.

## 2. Experimental Procedure

### 2.1. Coating Deposition

Single and bilayered RE coatings were deposited by RF (13.56 MHz) magnetron sputtering in a homemade system onto AA7075 and 6061 aluminum alloy substrates. The targets were 50.8 mm diameter CeO_2_ and La_2_O_3_ disks (Plasmaterials Company, Livermore, CA, USA) with purity of 99.99%. The targets were bound to copper backing plates to avoid rapid degradation and enhance the thermal conductivity. The system was evacuated by means of a rotatory and turbo pump to attain a residual pressure below 8 × 10^−4^ Pa. Before the deposition stage, the samples were cleaned by Ar etching and preheating of the substrates was done to reach the desired deposition temperature. The working pressure was 0.266 Pa and the distance between the magnetron guns and the samples was 60 mm. The discharge powers (*P*) for the RE thin films were set at 60 and 80 W, two preheating temperatures were tested (*T* = 80 and 200 °C) and the deposition time was changed as *t* = 25, 40, and 60 min. As for the RE bilayer coatings, the synthesis was carried out at 60 W for La and 80 W for Ce, using a temperature of 80 °C and 60 min of deposition. The synthesis was carried out under ultrahigh purity Ar atmosphere with a gas flow of 30 sccm.

An as received aluminum alloy AA7075 sheet (0.06 cm thick) was cut into 2 cm × 2 cm substrates whereas as obtained AA6061 bar was sliced into disks (2.5 cm in diam. and 0.2 cm thick). The coatings were simultaneously deposited onto glass and silicon (100) substrates, which were chosen for characterization purposes. Prior to deposition, the substrate surfaces were finely abraded using 1000, 1500, and 2000 grade SiC paper, cleaned by rinsing with deionized water, and finally sonicated in isopropyl alcohol and deionized water for 20 min, respectively.

### 2.2. Characterization of Samples

The phase composition and crystal structure of the as-synthesized films were determined by powder X-ray diffraction using an Advanced Bruker D8 diffractometer (Bruker, Billerica, MA, USA), with Cu K_α_ radiation at 35 kV and 25 mA, and at a scan rate of 0.021 min^−1^. Film morphology was examined by scanning electron microscopy using a JEOL JSM 7600F (JEOL Ltd., Akishima, Tokyo, Japan).

The topography and roughness were measured and analyzed by AFM (Veeco, Plainview, NY, USA), Veeco, Model diMultiMode V, controller diNanoScope V with cantilever rotated tips etched silicon probes (RTESP). The obtained data were analyzed using the NanoScope^®^ III version 5.12r3 software (Veeco, Plainview, NY, USA).

The chemical composition of the bilayered films was characterized by XPS (Thermo.com, East Grinstead, UK) measurements using a Thermo Fisher-VG system equipped with a dual X-ray source (Al–Mg) as well as hemispherical electron analyzer with seven channeltrons. In the experiments, the Al K*α* (1486.7 eV) source at 100 W was used. The data acquisition was carried out using a pressure of 7 × 10^−10^ Torr in the analysis chamber, and the resolution of the peaks was optimized using pass energy of 0.15 eV. The obtained data were analyzed using the SDP^®^ v4.1 software (International LLLC, Mountain view, CA, USA).

### 2.3. Electrochemical Performance

The corrosion behavior of coated and uncoated aluminum alloys was analyzed by open circuit potential (*E*_ocp_), polarization resistance (*R*p), potentiodynamic polarization and EIS measurements using a 3.0 wt % NaCl solution as corrosive medium. A potentiostat/galvanostat (Gamry Instruments, 600 series, Warminster, PA, USA) was used with a conventional experimental set-up of a three-electrode cell. A graphite bar (counter electrode) and a saturated calomel electrode (SCE, reference electrode) were employed to perform the corrosion experiments. The working electrode had an exposed area of 0.126 cm^2^. Polarization resistance measurements were conducted from 20 mV vs. SCE (cathodic) to 20 mV vs. SCE (anodic) of corrosion potential at a sweep rate of 0.5 mV s^−1^. To evaluate the susceptibility of the surface of the samples to pitting corrosion and to obtain information about the corrosion rate and corrosion potential, potentiodynamic polarization curves were scanned. These curves were measured from cathodic to anodic areas from −250 mV vs. SCE (*E*_ocp_) to 1000 mV vs. SCE at a sweep rate of 0.5 mV s^−1^. EIS measurements were carried out from the frequency region of 10^5^ to 0.01 Hz (10 frequency points per decade) with amplitude of mV rms. At least three replications were used for every corrosion-rate measurement.

## 3. Results and Discussion

### 3.1. Influence of Process Parameters on Ce and La Oxide Sputtered Coatings

To study the effects of power (*P*), substrate temperature (*T*) and deposition time (*t*) on the film composition as well as its corrosion behavior on AA6061 and AA7075 aluminum alloys, two sets of experiments were carried out. The first experiment series was realized maintaining a constant value of two variables and so on up to obtain a complete study of the influence of the deposition parameters. In this case, other deposition parameters were kept constant: 20 m Torr vacuum pressures, substrate-to-target distance (ds–t) was fixed at 6 cm and 30 sccm of argon. The second set of experiments was realized taking into account the best deposition parameters to produce Ce/La and La/Ce oxide bilayers coatings on both aluminum alloys and glass or silicon substrates for convenient characterization. Consequently, the diverse experiments were discussed in terms of the effect that the operating conditions have on the morphology, thickness, and/or electrochemical behavior of the CeO_2_ and La_2_O_3_ coatings deposited by RF magnetron sputtering on the AA6061 and AA7075 aluminum alloy.

#### 3.1.1. Structural Analysis of Sputtered Ce and La Oxide Coatings

Thin films of sputtered Ce and La oxide coatings as well as other sputtered thin films show a lateral variations along the substrates surface [36,37]; however, in this work, it was verified that this lateral variation is much smaller than the variation of the properties with power (*P*), substrate temperature (*T*) and deposition time. For this reason, metallic glass or silicon substrates were used for convenience characterization.

**Figure 1:**
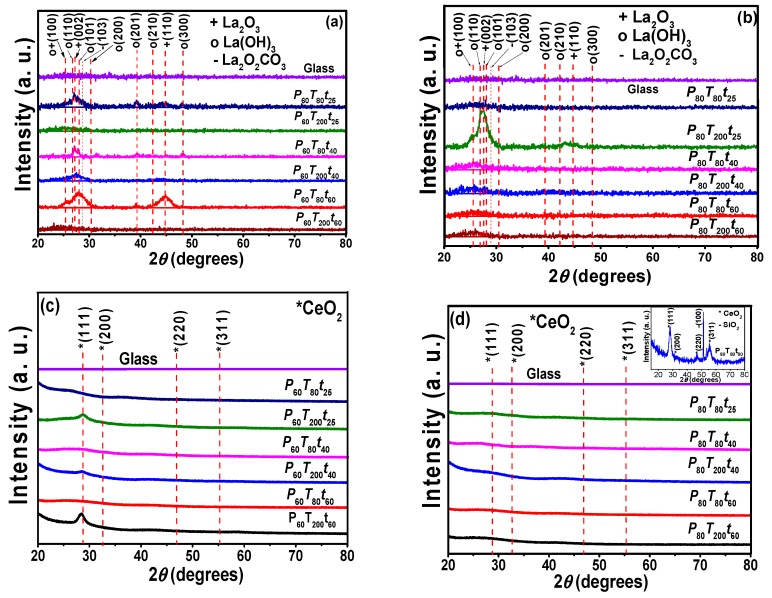
XRD patterns of sputtered (**a**) lanthanum coatings at constant power P = 60 W, (**b**) lanthanum coatings at constant power P = 80 W, (**c**) cerium coatings at constant power P = 60 W, (**d**) cerium coatings at constant power P = 80 W. The samples were deposited on glass substrates varying deposition time (25, 40 and 60 min) and substrate temperatures (80 and 200 °C).

Figure 1a–d shows X-ray diffraction patterns of the sputtered deposited RE coatings at different deposition parameters on glass substrates. XRD spectra show typical amorphous signal of glass substrates at low angles, ~12° (θ–2θ). The amorphous peak of the glass substrate diminished with the increased of thickness which in turn were quite dependent of the deposition parameters. In Figure 1a,b, the prevailing peaks matched well with the signals of La_2_O_3_ and La(OH)_3_ with a hexagonal structure (PDF 40–1281 and 36–1481). Under standardized scales, the crystalline structure of the samples presented important changes depending of the deposition parameters; such differences are particularly observed at the (100), (002), (101), and (110) planes of La_2_O_3_ and at the (100), (110), (101), (200), (201), and (300) planes of La(OH)_3_.

A magnification in the 20–60° range (θ–2θ) was performed in the as–prepared samples in order to observe and corroborate in detail crystallinity changes (see Figure S1, Supplementary Materials). The amplified spectra confirm the crystallinity of oxide/hydroxide lanthanum compounds, but it also shows small peak that seems to correspond to La_2_O_2_CO_3_ phase (PDF 48–1113). K. Kakushima et al. [38] reported that La_2_O_3_→La(OH)_3_ transformation accompanied with carbonates (La_2_O_2_CO_3_) can occur when La_2_O_3_ coatings were exposed to the atmosphere and in our case, due to the proposed applications of these coatings, the substrate/coating systems were not exposed to special atmosphere after deposition; whereby hydroxide and small carbonate signals were formed.

The amount of La(OH)_3_/La_2_O_2_CO_3_ in the film are a balance between the deposition parameters. The reactivity of La_2_O_3_ to form La(OH)_3_/La_2_O_2_CO_3_ films is also inhibited as the power was increased. Additionally, XRD patterns of La coatings fabricated at *P*_80_*T*_80_*t*_60_, indicate a preferential growth at the (100) plane. It is also evident a broadening of the main peaks as the deposition time and/or the power increased (Figure 1a,b). An estimation of the crystallite size under the different experimental conditions was determined using Scherrer equation for each peak regarding each RE coating [39].

(1) $$L_{hkl}=\frac{\lambda k}{\beta_{hkl}\cos\theta_{hkl}}$$

In this equation, *k* is the Scherrer constant (0.9 for sphere crystallites), *λ* is the X-ray wavelength (0.15418 nm), $\beta_{\mathrm{hkl}}$ is the full width at half medium (hkl) in radians of XRD peak, and $\theta_{\mathrm{hkl}}$ is the Bragg angle. The pseudo-Voigt profile function was used to determine the full-width and half-maximum broadening, considering symmetrical peaks for the fitting [40,41,42]. The crystallite sizes for lanthanum specimens varied from ~0.9 to 12.8 nm with a film thickness ranging from ~269.5 to 390.2 nm (60 W) and from ~499.0–835.5 nm (80 W), confirming the trend to diminish the crystallite size with the process parameters (Table 1).

The obtained results seem to be in good agreement with previous works [43,44], where it has been found that the crystallite size is reduced with the increasing power and deposition time, although it is also obvious that lattice strain, dislocations and grain boundaries or even instrumental contributions can add to the signal broadening. These results suggest that the deposition parameters can be modulated substantially to influence the final film microstructure.

On the other hand, Figure 1c,d shows the XRD diffraction pattern of Ce coatings from which it can be observed that independently of the substrate temperature and deposition time, preferred signals were detected from the (111) plane at 60 W. The growth of this orientation was more pronounced at high deposition time (60 min) and substrate temperature (200 °C). By increasing the power to 80 W, the coating exhibited a weak intensity of the fluorite cubic CeO_2_ structure at the (111) plane at about 28.52° (PDF 34–0394). A XRD of CeO_2_ coating deposited on Si (1000) at P_80_T_80_t_60_ and analyzed by grazing angle X-ray diffraction (3°) is shown as inset in Figure 1d. In this figure, it is seen nanocrystalline CeO_2_ films with orientation in the (111) and (311) planes. Ce films produced at 80 W showed nanocrystalline films whereas at 60 W, only at high temperature, oriented films were observed. The widening of peaks caused a small displacement in the (111) plane toward low diffraction angles, which can be correlated with the lattice expansion; during the Ce film formation, the Ce^3+^ and Ce^4+^ fractions can coexist, provoking a lattice deformation at the same time that the crystallite size decreased. In this case, the crystallite size forming crystalline films ranged from 1.1 to 7.0 nm, with a film thickness calculate from profilometry method of 12.7–40.1 nm (60 W) and 42.2 to 158.1 nm (80 W).

The increase thickness is closely related to the RF power density, which can be explained in terms of bombarding argon ions; i.e., the ceramic target with high energy at high target bias voltage, temperature, and deposition time.

#### 3.1.2. Nature and Chemical Composition of RE Films Deposited on Metallic Substrates

**Figure 2:**
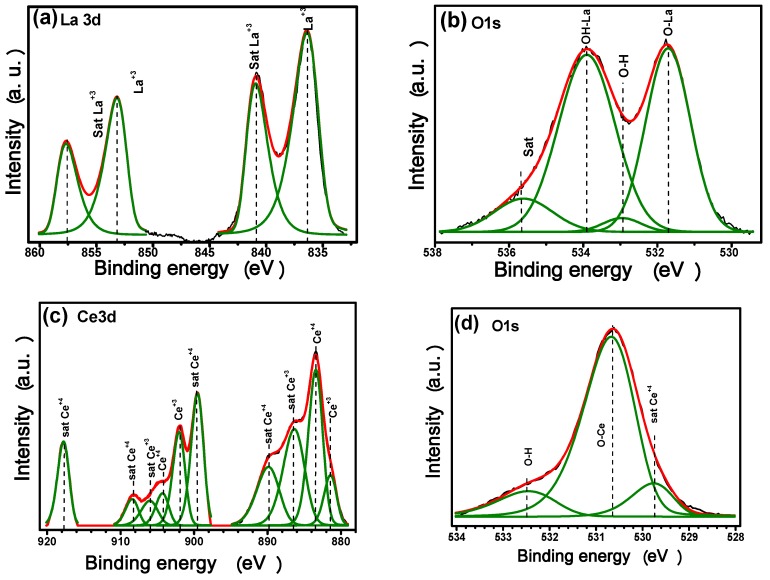
Representative high resolution XPS spectra of as-obtained samples at *P_60_T_80_t_60_*: (**a**) La 3d, (**b**) La O1s, (**c**) Ce 3d, and (**d**) Ce O1s.

We have previously shown that deposition parameters exert a strong influence in the rare earth composition which then conditioned the barrier properties of the coatings deposited on aluminum alloys [45,46]. In fact, during the process “metallic glasses” or amorphous films can be produced as a consequence of RE polymorphic transitions, predominantly observed for Ce [47]. In order to determine the nature and composition of the sputtered RE films on both aluminum alloys, XPS analysis were performed. Samples were measured at the same time on deposited metallic and silicon substrates. Low XPS resolution spectra of RE coatings on metallic substrates reveals that films are mainly composed of Al, Ce, La as well as significant amounts of O in the coatings outer layers (Figure S2, Supplementary Materials). In this figure, there is also a small signal of absorbed C from the air on the surface of the films. The high resolution spectra and their deconvolution on AA6061 substrates (as reference) are also displayed in the Supplementary Materials (Figure S3). The Al 2p shows the aluminum in the Al^3+^ and Al^0^ states with two components at binding energies of 74.4 eV, even in some conditions these signals were hard to split or deconvolute. To eliminate substrate interferences during quantification of the RE thin films, XPS analyses were realized on silicon; Figure 2a–d displays representative spectra of high resolution La3d, Ce3d and O1s core levels which were analyzed at the different experimental conditions.

The La3d core level spectra of lanthanum compounds show their well-known splits into 3d_5/2_ and 3d_3/2_ components (due to a spin–orbit interaction) with their characteristic doublet (Figure 2a). The peaks at 853.48 and 836.71 eV matched well with La^3+^ species. The corresponding satellite peaks are observed at 857.97 and 841.17 eV, respectively, with a separation of the main peak of Δ*E* ≈ 4 eV which is in good agreement with previous reports [48].

The O1s level spectrum was deconvoluted into two main peaks located at 532.01 eV and 534.45 eV, which are correlated with lanthanum oxide/hydroxide compounds, even the peak at ~534 eV could indicate the presence of La_2_O_2_CO_3_ (Figure 2b). The oxide/hydroxide ratio varied from 0.62 to 0.81 due to the well-known hygroscopic properties, (Table 2) [49,50]. The chemical environment around the O atoms indicates that under the synthesis parameters, the obtained films are free of contamination or foreign compounds such as La_2_O_2_CO_3_. However, the lanthanum hydroxides or carbonates could be readily formed when the film is exposed to the atmosphere.

Two features can be highlighted in this analysis. Firstly, the deconvoluted spectra show that both Ce^4+^ and Ce^3+^ chemical states were present for the sputtered coatings, although Ce^4+^ species predominate. Secondly, the XPS results confirmed the formation of both Ce^4+^ and Ce^3+^ species during the sputtering process, but the XRD peaks corresponding to the crystalline Ce_2_O_3_ hexagonal phase were missing; thus, in agreement with a previous work, it can be suggested that Ce^3+^ species may exist as Ce_2_O_3_ in an amorphous phase or around oxygen vacancies in CeO_2_ [51,52,53,54].

O1s XPS spectra have been a useful tool to differentiate molecular water from hydroxyl dissociated groups on the surface of ceria compounds (Figure 2d). The three features in our spectra located at 530.06, 530.94 and 532.66 eV have been assigned to lattice oxygen, hydroxyl groups and adsorbed water, respectively [55].

#### 3.1.3. Morphological and Topographical Properties of RE Thin Films on Metallic

**Figure 3:**
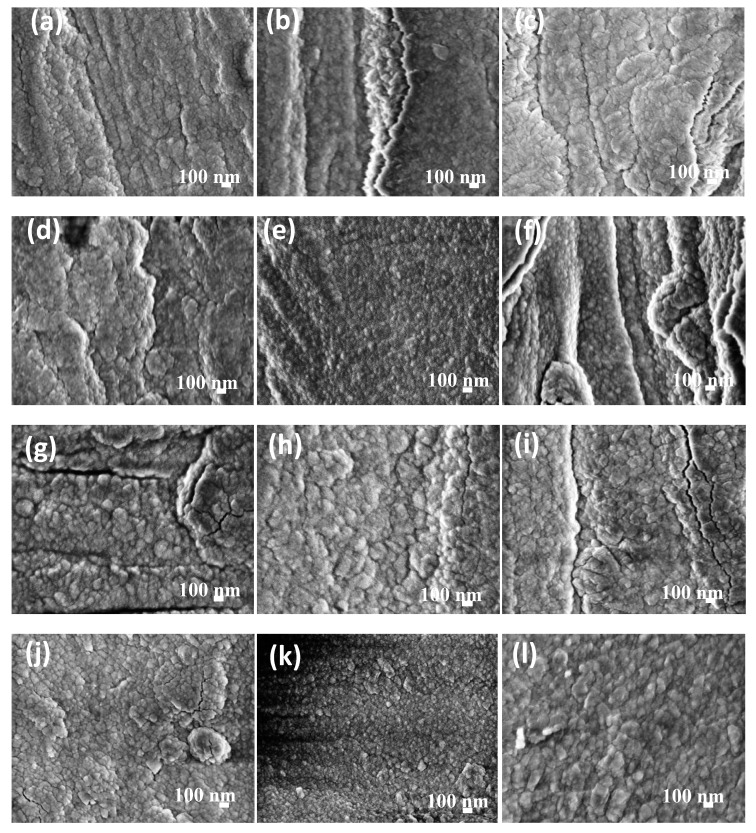
High Resolution Scanning Electron Microscopy (HRSEM) images of La coatings on AA6061 aluminum alloy substrates: (**a**) *P_60_T_80_t_25_*, (**b**) *P_60_T_80_t_40_*, (**c**) *P_60_T_80_t_60_*, (**d**) *P_60_T_200_t_25_*, (**e**) *P_60_T_200_t_40_*, (**f**) *P_60_T_200_t_60_*, (**g**) *P_80_T_80_t_25_*, (**h**) *P_80_T_80_t_40_*, (**i**) *P_80_T_80_t_60_*, (**j**) *P_80_T_200_t_25_*, (**k**) *P_80_T_200_t_40_*, and (**l**) *P_80_T_200_t_60_*.

**Figure 4:**
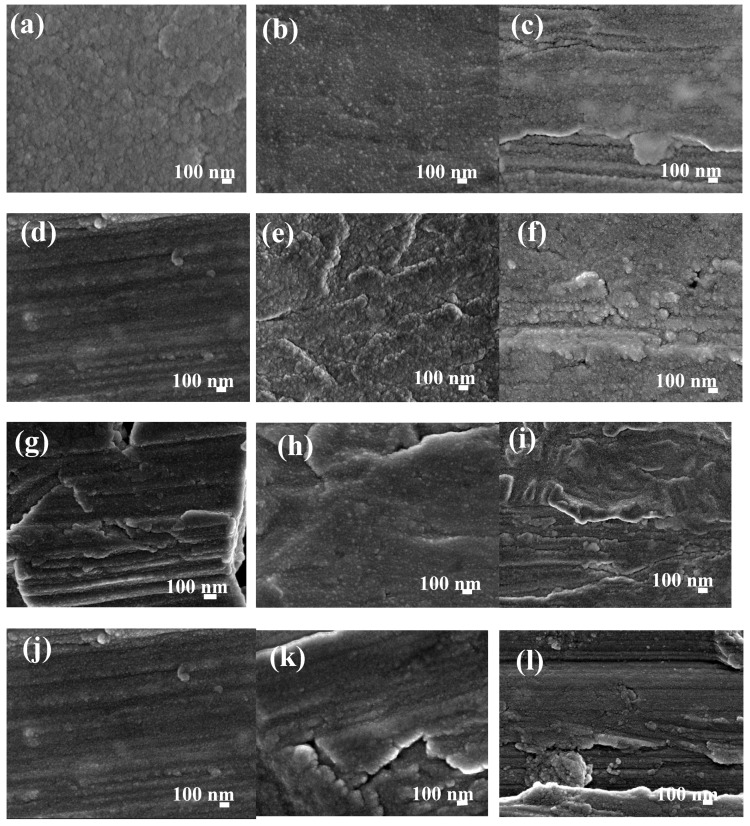
High Resolution Scanning Electron Microscopy (HRSEM) images of Ce coatings on AA6061 aluminum alloy substrates: (**a**) *P_60_T_80_t_25_*, (**b**) *P_60_T_80_t_40_*, (**c**) *P_60_T_80_t_60_*, (**d**) *P_60_T_200_t_25_*, (**e**) *P_60_T_200_t_40_*, (**f**) *P_60_T_200_t_60_*, (**g**) *P_80_T_80_t_25_*, (**h**) *P_80_T_80_t_40_*, (**i**) *P_80_T_80_t_60_*, (**j**) *P_80_T_200_t_25_*, (**k**) *P_80_T_200_t_40_*, and (**l**) *P_80_T_200_t_60_.*

The microstructure and morphology of sputtered RE coatings onto AA6061 substrates are shown in Figure 3 and Figure 4a–l. The SEM images show typical RE growth of nanocrystalline coatings; although, the examination of the coating microstructures could not determine significant difference in the growth film with the synthesis parameters. In general, the initial stages for the sputtered layer build-up was observed as a rapid deposition on the surface in the form of islands of different sizes; as the deposition time was increased, the initial islands grew and merged to produce a thin film that covered the entire substrate. In contrast with our previous reports, where a uniform and crack-free film was observed on the AA6061 aluminum alloys, some cracks are observed in the coatings [45]. Micro-sized defects imply nucleation and island growth process; however, in both substrates was observed that by increasing the coating thickness, the quantity and depth of these defects decreased as a consequence of island coalescence.

Other small differences were observed with the increasing of power and deposition time. The small agglomerates that conform the film changed from fine to coarse with a film thickness that varies from 269.5 nm to 835.5 nm and from 19.3 nm to 158.1 nm for La_2_O_3_/La(OH)_3_/La_2_O_2_CO_3_ and CeO_2_/Ce_2_O_3_ coatings, respectively (Table 1). It is also evident that under similar deposition conditions, low thicknesses are obtained with cerium in comparison with lanthanum coatings. It is well-known that the thickness of RE coatings and its electrochemical performance determinates the economic profitability, which also depends on the adhesion and roughness of the obtained films. Atomic force microscopy has been widely used in coating materials to determinate growth film features and roughness. Representative set of AFM images from sputtered cerium and lanthanum oxide deposited on AA6061 substrates, as well as the AFM images for metallic substrates are shown in the Supplementary Materials (Figure S4). Ce oxide/hydroxides coatings displayed a trend to reduce the surface roughness with the deposition time and temperature in the chamber with Ra values. The rougher surface was obtained at *P_80_T_80_t_25_* (289.0 nm), and reduces with the time to 127.9 nm. Smoother Ce coatings of 59.6 nm were obtained at *P_60_T_2000_t_60_*. On the other hand, topography of La oxide coatings did not shown a clear trend in the *R*_a_ values with the deposition parameters (Table 1)*.* The lanthanum coatings show agglomerates forming the film, which vary from 50 to 300 nm in diameter with a height of ~20–55 nm. In this case, the Ra values were between 36.1–166.0 nm. The differences in the *R*a can be explained in terms of the microdefects observed in the SEM and AFM images which occurred when energetic ions were accelerated by the voltage discharged toward the substrate, this phenomenon in turns provokes re-sputtering at the growing coating surface. The re-sputtering can explain the low thickness and rougher surface of the Ce coatings in comparison with La films deposited at similar deposition parameters.

### 3.2. Microstructural Analysis of Ce/La and La/Ce Oxide Bilayered Coatings

**Figure 5:**
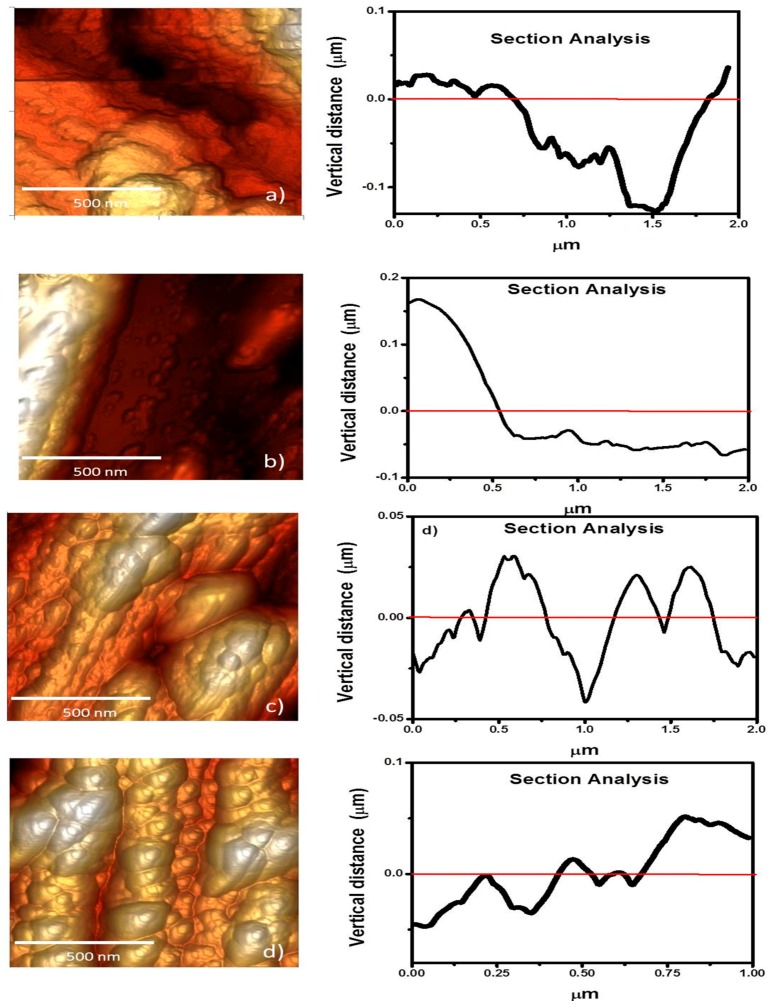
Representative 3D images of sputtered bilayered films at 80 °C and 60 min: (**a**) Ce_80W_/La_60W_, (AA6061), (**b**) Ce_80W_/La_60W_ (AA7075), (**c**) La_60W_/Ce_80W_, (AA6061), and (**d**) La_60W_/Ce_80W_ (AA7075). The section analysis is also shown as a height profile.

From the overall conditions and microstructural characterization of Ce and La layers, Ce/La and La/Ce oxide bilayered coatings were produced at *P*_80_*T*_80_*t*_60_ and *P*_60_*T*_80_*t*_60_ on both aluminum alloy substrates. These conditions were selected from initial electrochemical performance of the individual coatings. The topographical height profiles of bilayered coatings were analyzed by AFM analysis and shown in Figure 5a–d. It is important to mention that the analysis considered other projections to confirm the height profile. In general, the bilayered coatings showed closed aggregates or domains with a separation between them of barely 20 nm, and are ~130 nm in height. The average base roughness is clearly modified depending on the RE element that is deposited onto the metallic substrate; i.e., aluminum alloy/La(*P*_60_*T*_80_*t*_60_)/Ce(*P*_80_*T*_80_*t*_60_) and the RE layers were smoother than those observed in the aluminum alloy/Ce (*P*_80_*T*_80_*t*_60_)/La (*P*_60_*T*_80_*t*_60_) system (Table 1). The domain distribution of the lanthanum films appears non-uniformly and it seems that they tend to grow from the center to the edges. Macrodomains appear as bigger aggregates of polymorphic nanostructures in size and shape. The layer-by-layer growth mechanism of the mixed oxides shows a height from crest to valley of about 11 nm.

**Figure 6:**
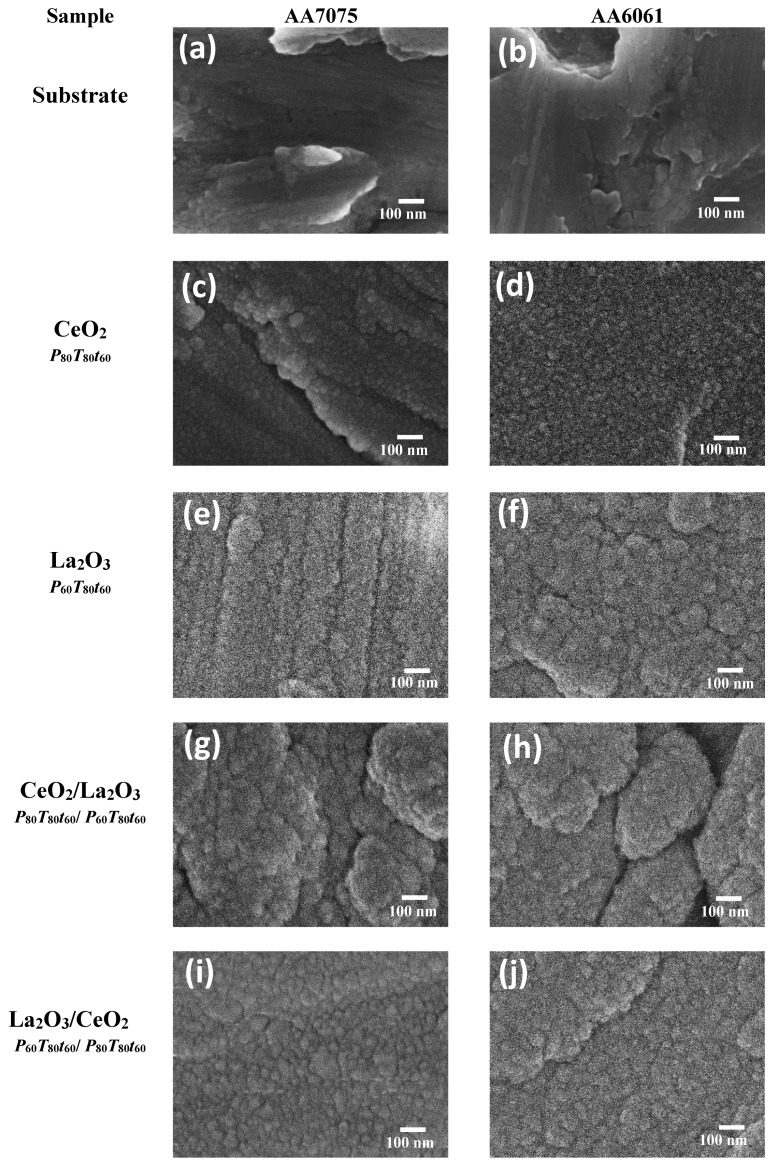
Typical High Resolution Scanning Electron Microscopy (HRSEM) images of oxide sputtered bilayered coatings, their corresponding comparison with pure coatings and bare aluminum. (**a**) Uncoated AA7075, (**b**) uncoated AA6061, (**c**) AA7075/Ce (*P_80_T_80_t_60_*), (**d**) AA6061/Ce (*P_80_T_80_t_60_*), (**e**) AA7075/La (*P_60_T_80_t_60_*), (**f**) AA6061/La (*P_60_T_80_t_60_*), (**g**) AA7075/Ce/La (*P_80_T_80_t_60_/P_60_T_80_t_60_*), (**h**) AA6061/Ce/La (*P_80_T_80_t_60_/P_60_T_80_t_60_*), (**i**) AA7075/La/Ce (*P_60_T_80_t_60_/P_80_T_80_t_60_*), and (**j**) AA6061/La/Ce (*P_60_T_80_t_60_/P_80_T_80_t_60_*).

The morphology of the bilayered samples was also analyzed by SEM measurements and the results are shown in Figure 6a–j. SEM micrographs revealed a dense fine-grained morphology in all the systems deposited on both aluminum alloys. The morphology did not change with the order of deposited (Ce (*P*_80_*T*_80_*t*_60_)/La(*P*_60_*T*_80_*t*_60_) or (La(*P*_60_*T*_80_*t*_60_)/(Ce (*P*_80_*T*_80_*t*_60_) layers, growing from islands up to covering the entire substrate surface with an average grain size of about 15 nm. The resulting coatings displayed a film thickness of 268.6 nm (at deposition rate of Ce/La; 0.075 nm s^−1^) and 284.3 nm (La/Ce; 0.079 nm s^−1^) for AA6061 substrates whereas the thickness on the AA7075 substrates was 182.1 nm (Ce/La; 0.050 nm s^−1^) and 193.1 (La/Ce; 0.054 nm s^−1^). The deposition rate was lower (~30%) when AA7075 was used as substrate, this variation can be correlated with the composition and features of the oxide film formed on the substrate surface. Generally, the mobility of atoms deposited on the substrate surface must have the adequate energy to be linked together, forming initial grains for island shape, and extend to the substrate. Thus, the necessary conditions for favoring the growth of energetic atoms as stable grains, and increasing the growth rate of the layer are favored with the AA6061 aluminum alloy.

**Figure 7:**
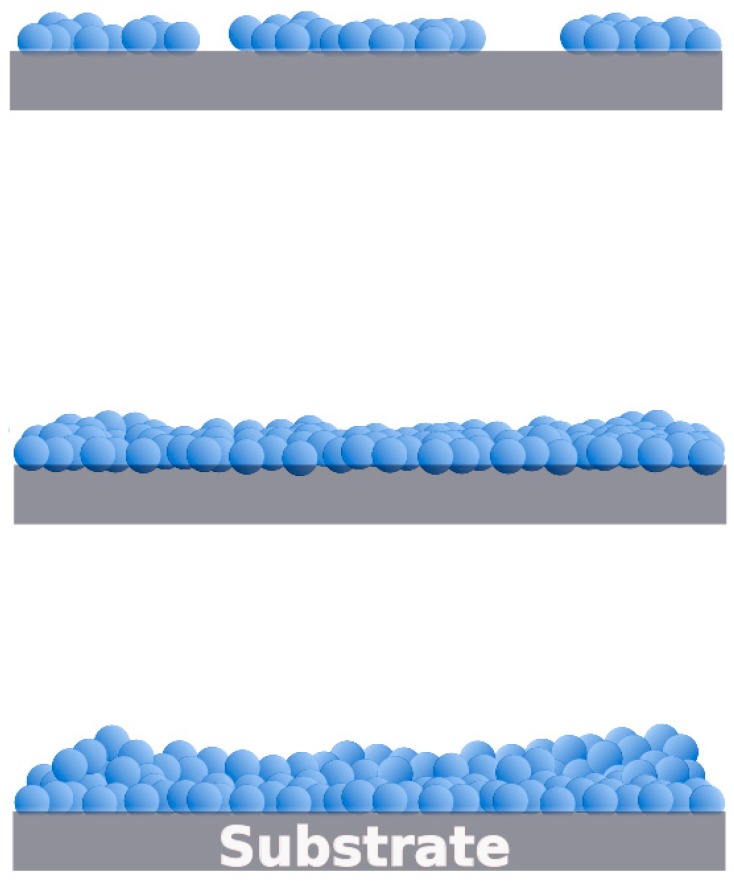
Growth film pathway (Stranski–Krastanov) characteristic of bilayered RE coatings.

Additionally, it is well recognized that an atomic process on solid substrate in different steps governs the nucleation and growth of thin films: (1) adsorption at special site, (2) surface diffusion, (3) nucleation and (4) inter-diffusion. The surface diffusion involves a condensation and re-evaporation which occur by basic film growth modes: Volmer–Weber (island), Frank–van der Merwe (layer) and Stranski–Krastanov (island-layer). The last one is related to the accommodation of elastic strain associated with epitaxial lattice misfit whereas the former processes are easily understood on the basis of macroscopic wetting [56,57]. During the film growth, surface roughening plays an important role since reduces the average film strain provoking that islands are more free to expand or contract deforming the vicinity leading the island repulse each other. When the strain accumulated is higher than surface energy, the 2D to 3D transition take place, supporting the strain relaxation at time that facilitate the nucleation of dislocations. High thickness and roughness in the coatings are commonly favored through the Stranski–Krastanov mechanism [58,59]. From AFM and profilometry measurements, it is clear that under our experimental conditions the film growth was not modified by the order of the RE deposition and seems to follow a typical Stranski–Krastanov mechanism (Figure 7).

**Figure 8:**
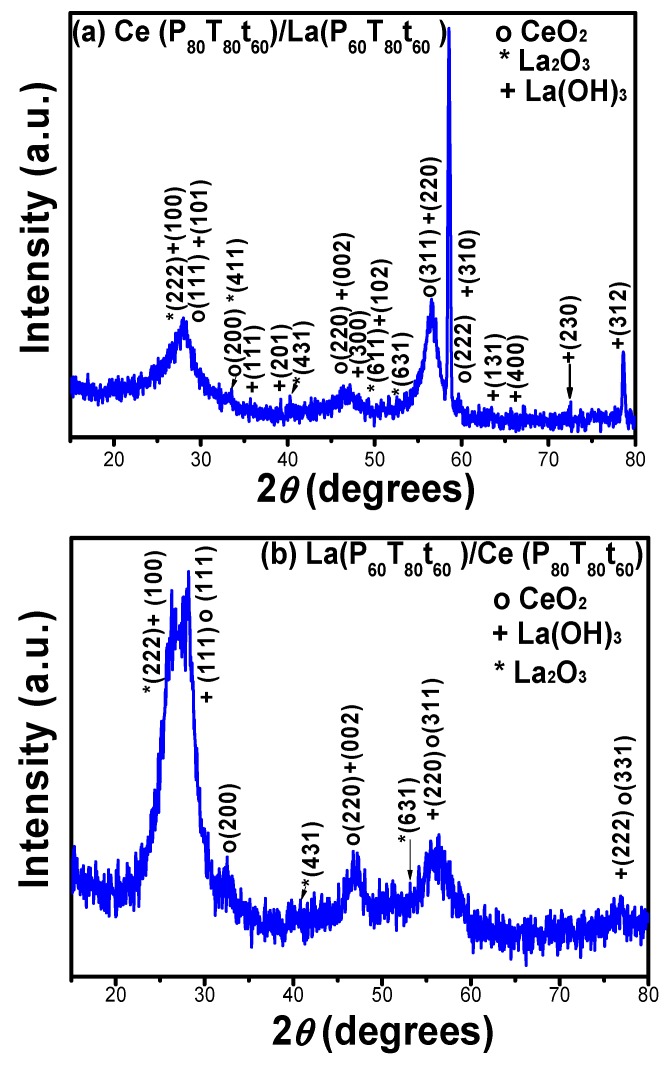
XRD of RE oxide bilayered coatings: (**a**) Ce(*P_80_T_80_t_60_*)/La(*P_60_T_80_t_60_*) and, (**b**) La(*P_60_T_80_t_60_*)/Ce(*P_80_T_80_t_60_*) onto Si (100) substrates.

In order to correlate the development of the bilayered microstructure with the coating thickness, X-ray diffraction patterns of sputtered samples were analyzed on Si (100) substrates and the spectra are shown in Figure 8a–b. The XRD patterns show a combination of the RE oxide compounds with their respective structural phase. The average crystallite size for Ce/La coatings was ~16.84 nm whereas La/Ce films show a crystallite size of ca. 2.9 nm. The crystallite size and roughness of bilayered coatings was slightly enhanced in comparison with monolayers. Considering that the ion bombardment and temperature on the growing coating surface would induce compressive internal strain by causing atomic displacements and densification of the coating, the compressive strain associated with crystallite coalescence would be more evident during the substrate/La/Ce interaction [33].

### 3.3. Electrochemical Measurements

#### 3.3.1. Potentiodynamic Curves

It is known that during the corrosion test of aluminum, a faster generation of Al^3+^ at anode sites resulted in a reduction of the local pH, which promotes gas evolution. Hydrogen gas evolution can be used to position continuous localized corrosion [60,61]. It is also important to note that small changes in the microstructure and/or in the passivating Al_2_O_3_ film could be provoked during the sputtering of RE films at 80 and 200 °C. However, in this work, it was only analyzed the influence RE deposition parameters and the barrier properties that provided in aggressive media [62,63,64,65].

In order to assess the passivation behavior of coated AA7075 and AA6061 aluminum substrates, potentiodynamic tests were performed in a 3.0 wt % NaCl solution. Potentiodynamic techniques are based on the application of a time dependent potential over the working electrode and the current response is measured. Specifically for corrosion studies, polarization scans are used to determinate typical anodic regions and the reaction that can be accompanied in dependence of the potential. Features of the curve such as open circuit potential, active region range, passivation potential, passive region, breakaway potential (localized breakdown of passivity) can help to determinate the thermodynamic and kinetic information. In the cathodic region it can be commonly established the region of oxygen reduction reaction, changes in the reaction pathway from control of charge transfer resistance process to diffusion process, and finally the water reduction reaction.

**Figure 9:**
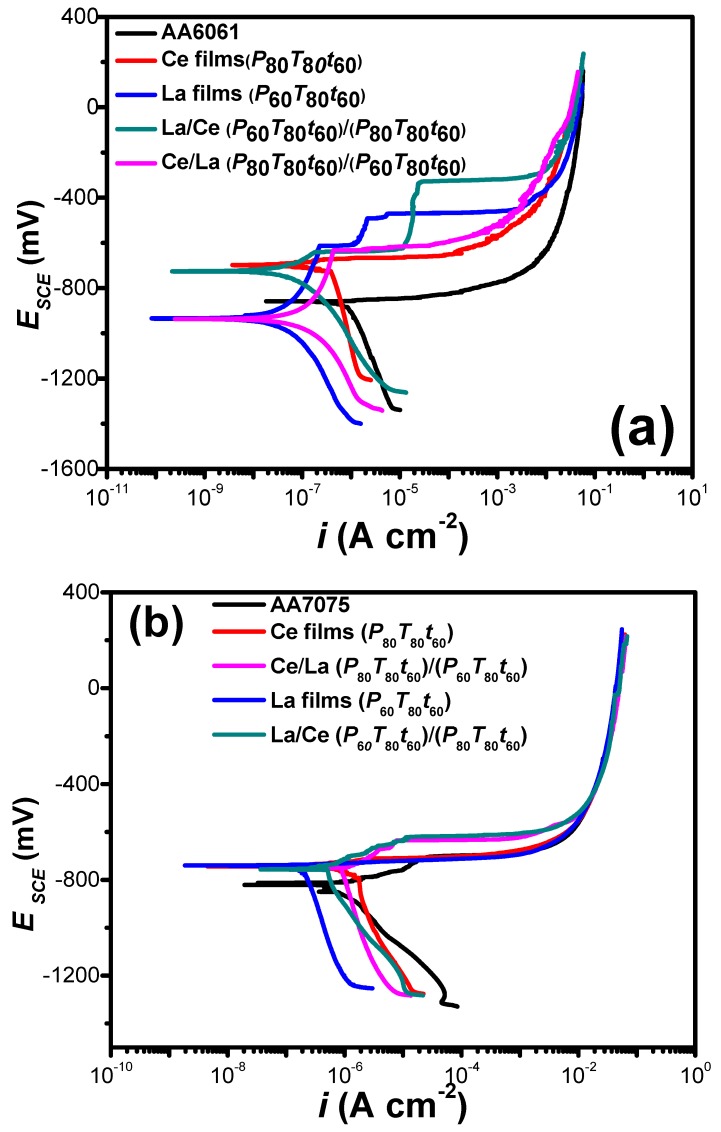
Polarization curves of RE coatings synthesized at 60 or 80 W (*T* = 80 °C and *t* = 60 min) and bilayered coatings on: (**a**) AA7075 and (**b**) AA6061 aluminum alloys.

La/Ce bilayered coatings fabricated at *P*_80_*T*_80_*t*_60_ and *P*_60_*T*_80_*t*_60_ on both aluminum alloy substrates are shown in Figure 9a,b. For comparison purposes, the electrochemical performance of individual Ce or La oxide coatings, under similar deposition conditions, and bare substrates are also shown in the plot.

In general, the potentiodynamic curves showed a positive displacement shifting the corrosion potential (*E*_corr_). The displacement of *E*_corr_ is because of the decrease or increase in the evolution rate of O_2_ reduction and/or H_2_ production and the increase in the passivation current density (*i*_passive_). In the cathodic branch of both aluminum alloys, a region of mixed kinetic control is seen, where the current density tends to reduce by modifying the charge transfer mechanism. The performance of RE coatings appeared totally different when the polarization curves were recorded on different metallic substrates, highlighting the influence of substrate composition and its microstructures on the electrochemical performance. From Figure 9a,b, it is clearly seen that the RE coatings can accelerate or decelerate the O_2_ reduction/H_2_ evolution rate and decrease the *i*_passive_ value, and consequently shifts *E*_corr_ depending on the metallic substrate used.

In the anodic branch, uncoated aluminum (AA6061 and AA7075) showed two typical regions. In the first region, the dissolution of the aluminum alloy occurs above its open circuit potential because an active electrochemical reaction takes place on the surface and the anodic current increases rapidly from −863 to −240 mV*_SCE_* and from −806 to −90 mV*_SCE_*, for AA6061 and AA7075, respectively (Table 3). In a subsequent step, there is a passivation region beginning at potentials of −90 and −240 mV*_SCE_*. Sputtered La, La/Ce and Ce/La oxide coatings on AA6061 showed a tendency to form a passive stage with a current density of 1.65 × 10^−6^ A cm^–2^ (−611 mV*_SCE_*), 1.69 × 10^−5^ A cm^−2^ (−540 mV*_SCE_*), and 2.32 × 10^−7^ A cm^–2^ (−850 mV*_SCE_*), followed by a transpassive zone while the other systems remained with the shape of bare the aluminum.

On the other hand, Ce, La, Ce/La, and La/Ce oxide coatings on AA7075 substrates showed similar shapes in the anodic and cathodic branches. As it can be seen, in all the cases, no passive behavior occurred. The corrosion potential of the samples is about −740 mV*_SCE_* and does not show significant changes. The potential of coated samples is approximately 70 mV*_SCE_* more positive than the uncoated substrate. In the anodic branch, a strong increase in the current density with the potential is observed, suggesting that even with coating, the samples have pits at discrete locations on the surface; thereafter, a tendency to the repassivation of microsize pits can be observed [65]. It is known that during the growing process of an occluded pit, the concentration of metallic cations increases gradually due to the active dissolution within the pit [66,67,68,69]. Once the saturated concentration is reached, a salt film will be formed at the bottom of the pit. The dissolved metallic cations move outward through the salt film under the action of an electric field across the film. The stronger the field, the faster the metallic cations move through the film. Hence, at this stage, the growth of the pit is controlled by the ohmic potential drop across the salt film. The absence of a passive plateau suggested a limited effect of the RE coatings on the inhibition of the anodic dissolution of AA7075. However, the corrosion potential shifting to positive potentials suggests a certain protection degree.

The observed differences in both substrates can be explained as follows: it is well known that microstructures developed in high-strength aluminum alloys such as AA7075 are complex and incorporate a combination of equilibrium and nonequilibrium phases. Typically, such alloys have a chemical composition incorporating up to ten alloying elements. Such elements primarily include Zn, Mg, and Cu; however, appreciable and specific amounts of Fe, Si, Cr, Ti, Zr, and Mn are often present (both as deliberate additions and as impurities). As for localized corrosion, the intermetallics of particular interest are those that appear at the highest proportion, either by size or by frequency. For AA7075, such particles have been identified (in random order) as Mg_2_Si, MgZn_2_, Al_7_Cu_2_Fe, Al_2_CuMg, Al_2_Cu, and Al_3_Fe_25_ [70,71,72]. On the other hand, different precipitates have been identified in the AA6061 aluminum matrix such as Al_3_Fe, *α*-(Fe, Cr, Mn)_3_SiAl_12_, *β*-(Fe, Cr, Mn)_2_Si_2_Al_9_, Si, Mg_2_Si and *π*-(Fe, Cr, Mn) Mg_3_Si_6_Al_8_. In this context, although several factors affect aluminum dissolution, the substrate composition conditioned the microstructure, which in turn plays an important role in enhancing the passivation in aluminum alloys.

Kinetic parameters and corrosion rate of the as obtained samples are shown in Table 3. It is important to note that only Tafel slopes with a physical significance were calculated and shown in this table. The presence of deposited REs provoked changes in the anodic Tafel slopes, which varied from 93.9 to 128.2 and 41.3 to 92.1 mV dec^−1^, for AA 6061 and AA 7075, respectively. The lowest corrosion current densities were obtained for the sputtered La coatings (*P_60_T_80_t_60_*) onto AA6061 substrates (17.7 nA cm^−2^) followed by La coatings under the same deposition parameters onto AA7075 specimens (78.4 nA cm^−2^). In the case of RE bilayered coatings, the La/Ce bilayered films synthesized at (*P*_60_*T*_80_*t*_60_)/(*P*_80_*T*_80_*t*_60_) onto AA6061 substrates (99.1 nA cm^−2^) were more prominent than those presented by AA7075 samples under similar conditions (566.6 nA cm^−2^). The cathodic inhibition when La is deposited onto the metallic surface is consistent with previous works obtained by chemical methods. The results confirm that the La deposition prior to Ce films on both metallic substrates allowed to obtain less-imperfect coatings with a small crystallite size (~2.9 nm). Thicknesses of La/Ce films, 284.3 nm for AA6061 and 193.1 nm for AA7075 in comparison with Ce/La (268.6 nm and 182.1 nm) are also in good agreement with this observation.

In addition, it is also recognized that the charge mobility between the metal surface and the coating-electrolyte interface could also be suppressed with the increase in coating thickness, limiting the kinetics of the cathodic reactions [43,73,74]. Although coating delamination was not observed during the potentiodynamic measurements, the increase in internal stress-related defects during the sputtering process could also contribute to the deterioration during the corrosion evaluation. The susceptibility of the coated aluminum alloys with RE compounds using the sputtering technique in a chloride-containing solution toward the pitting attack was enhanced in the following order: AA7075/Ce (*P*_80_*T*_80_*t*_60_), 1440.6 nA cm^−2^ > AA7075/Ce/La (*P*_80_*T*_80_*t*_60_)/(*P*_60_*T*_80_*t*_60_), 949.8 nA cm^−2^ > AA7075/La/Ce (*P*_60_*T*_80_*t*_60_)/(*P*_80_*T*_80_*t*_60_), 566.6 nA cm^−2^ > AA6061/Ce (*P*_80_*T*_80_*t*_60_), 524.5 nA cm^−2^ > AA6061/Ce/La (*P*_80_*T*_80_*t*_60_)/(*P*_60_*T*_80_*t*_60_), 195.4 nA cm^−2^ > AA6061/La/Ce (*P*_60_*T*_80_*t*_60_)/(*P*_80_*T*_80_*t*_60_), 99.1 nA cm^−2^ > AA7075/La (*P*_60_*T*_80_*t*_60_), 78.4 nA cm^−2^ > AA6061/La (*P*_60_*T*_80_*t*_60_), 17.7 nA cm^−2^. An approximation of the inhibition efficiency was performed using the relationship $IE\left(\%\right)=\left(1-i_{\mathrm{corr}}/i_{\mathrm{corr}}^{o}\right)\times 100$, where $i_{\mathrm{corr}}$ and $i_{\mathrm{corr}}^{o}$ are the corrosion current densities of coated and uncoated samples. Even with the anodic dissolution characteristic of aluminum alloys, La coatings displayed inhibition efficiency (IE) above 95%.

#### 3.3.2. EIS Measurements

EIS is a quantitative tool to assess the corrosion protection afforded by different kinds of coatings; thus, it has been used to evaluate sputtered RE coatings on commercial aluminum alloys [45]. For comparison purposes, impedance data obtained for the bare substrates are also shown in the plots.

**Figure 10:**
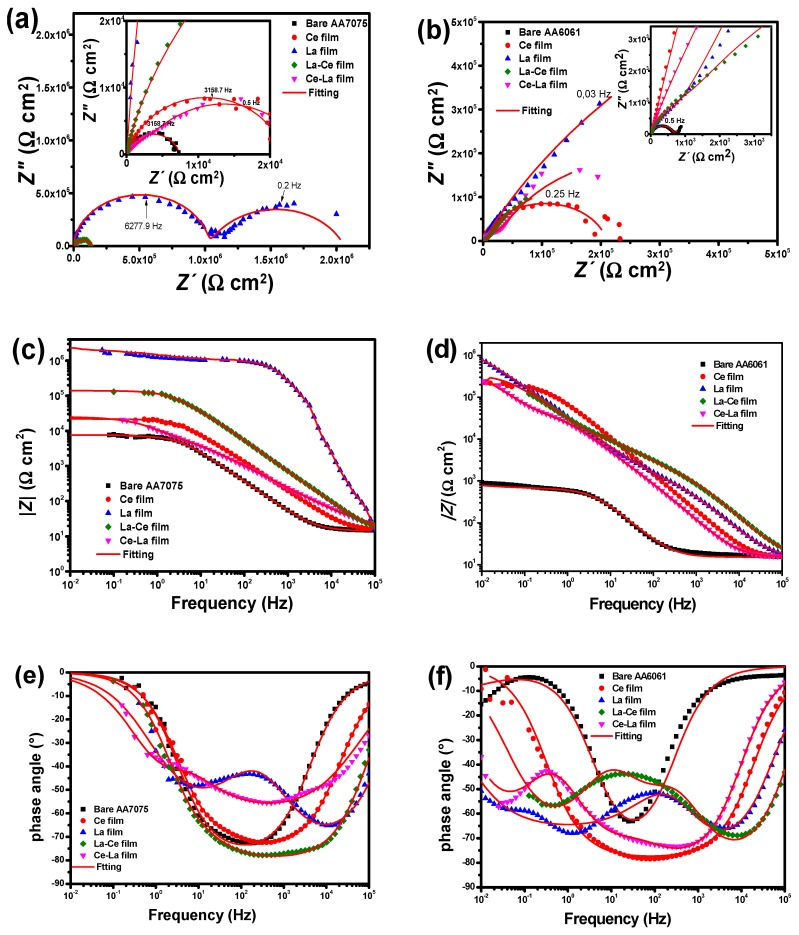
Nyquist and Bode spectra of sputtered coatings on (**a**–**c**) AA7075 and (**d**–**f**) AA6061 evaluated in a 3 wt % NaCl solution.

Figure 10a–f reports the EIS spectra of pure coatings and bilayered samples on both aluminum substrates in a 3 wt % NaCl solution. Considering that Nyquist and Bode plots exhibit an expected behavior, an increase in the total resistance with respect to the bare sample can be seen for all the coated samples with different magnitude orders depending on the substrate composition and deposition parameters. There are partially superimposed semicircles corresponding to two or three time constants. The changes in the shapes of the EIS spectra, total resistance, and width of capacitive semicircles are associated with differences in the film roughness and thickness, which conditioned the interaction between the electrolyte and RE coatings and eventually with the metallic substrate, i.e., the path that allows the solution to interact with the coating through the channels and in time reach the metallic substrate. The highest impedance values were obtained for La coatings on both substrates with *R*p values around 2.1 × 10^6^ Ω cm^2^ for AA7075 (*P*_60_*T*_80_*t*_60_) and 2.3 × 10^6^ for AA6061 (*P*_60_*T*_80_*t*_60_). The phase angle values remained very close to 80°, suggesting the formation and growth of a passive film. The order of the corrosion inhibition for sputtered RE coatings was La > La/Ce > Ce/La > Ce for AA7075 whereas for coated AA6061 substrates the provided protection was in the La > La/Ce > Ce/La > Ce order. The classical reactions for bare aluminum corrosion involve the metallic dissolution Al^3+^ + 3e^−^ → Al while cathodic reactions are O_2_ + 2H_2_O + 4e^−^ → 4OH^−^ and/or 2H_3_O^+^ + 2e^−^ → 2H_2_O [75,76,77]. On the other hand, for coated specimens with Ce films in contact with a NaCl solution can be dissolved as follows [78,79,80]:

(2) $$CeO_{2}+2H_{2}O\to Ce^{4+}+4OH^{-}$$

(3) $$Ce_{2}O_{3}+3H_{2}O\to 2Ce^{3+}+6OH^{-}$$

The cerium oxide compounds are dissolved in an aqueous medium with the subsequent production of OH^−^ ions. Ce^4+^ can be reduced to Ce^3+^, increasing the local pH value through the following reaction:

(4) $$Ce^{4+}+e^{-}\to Ce^{3+}$$

Then, the inhibition mechanism of the corrosion process occurs due to the formation of dense insoluble hydroxide deposits on the metallic surface:

(5) $$Ce^{3+}+3OH^{-}\to Ce{\left(OH\right)}_{3}$$

or

(6) $$Ce^{4+}+4OH^{-}\to Ce{\left(OH\right)}_{4}$$

(7) $$\;4Ce^{3+}+O_{2}+4OH^{-}+2H_{2}O\to 4Ce{\left(OH\right)}_{2}^{2+}$$

Eventually, the formation of CeO_2_ on the metallic substrate can be possible, providing the so-called self-healing properties of the ceria thin film:

(8) $$Ce{\left(OH\right)}_{2}^{2+}+2OH^{-}\to CeO_{2}+2H_{2}O$$

or

(9) $$Ce{\left(OH\right)}_{4}\to CeO_{2}↓\;+\;2H_{2}O$$

On the other hand, La coatings in the aqueous medium can also be dissolved, which propitiate an alkaline environment that leads to the precipitation of lanthanum hydroxides [81,82]:

(10) $$La_{2}O_{3}+3H_{2}O\to 2La^{3+}+6OH^{-}$$

(11) $$La^{3+}+3OH^{-}\to La{\left(OH\right)}_{3}↓$$

The lanthanum hydroxides undergo a hydrated process, resulting in the formation of oxide elements, passivating again the surface:

(12) $$2La{\left(OH\right)}_{3}\to La_{2}O_{3}+3H_{2}O$$

**Figure 11:**
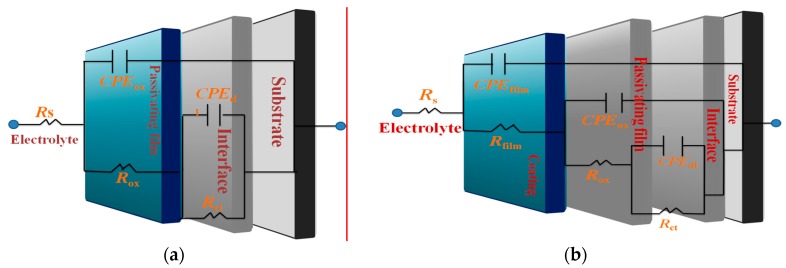
Equivalent circuits used for fitted experimental EIS data of RE coated metallic substrates. (**a**) bare substrates (**b**) coated specimens.

The interpretation of the measured data for *Z*(*ω*) was carried out by their comparison with the predictions of the theoretical model based on equivalent circuits, and the parameters were established by best fitting of the theoretically calculated impedance plots to experimental ones with a chi-square (*χ*^2^) < 10^−3^. The fitting of experimental data was accomplished using the appropriate electrical equivalent circuits that are shown in Figure 11a-b. The model fitted to the bare substrates with a natural oxide layer has been previously proposed by Jütner [83] and it has been used as a reference for the fitting of AA7075 and AA6061 substrates. In the electrical equivalent circuit, *R*_s_ represents the solution resistance; *R*_ox_ is the oxide layer associated resistance; *R*_ct_ is the charge transfer resistance; *CPE*_ox_ is a constant phase element (*CPE*) related to the electrical oxide film capacitance; and *CPE*_dl_ represents the constant phase element of the double layer. In addition to the previous electrical elements, the equivalent circuits for coated samples included *R*_film_, coating resistance or pores and *CPE*_film_, used to model the frequency dependence of electrochemical phenomena on the coating. The *CPE* elements were used instead of an ideal capacitive element due to deviation from an ideal dielectric behavior. The capacitance of *CPE* can be expressed as follows [84]:

*Z*_CPE_*=* −1/(*Y*_o_(*jω*)*^n^*) (13)

where $j=\sqrt{-1}$ is the imaginary unit, ω=2πf is the angular frequency (in rad s^−1^), f is the frequency in hertz and n is the dimensionless empirical exponent corresponding to phase deviation. When *n* = 1, the system behaves like a pure capacitor and *CPE* = *C*. Then, capacitance dispersion is associated with different levels of heterogeneities in the interfacial region.

The fitting parameters confirms that the high values of the polarization resistance are obtained with lower surface roughness as a result of the small crystallite size mainly obtained at *P*_60_*T*_80_*t*_60_ for La films (Table 4).

The value suggested that RE coatings on aluminum alloys promote a stabilization of the oxide layer and consequently, high values of *R*_ct_ are observed. This characteristic is clearly related to a combination of the surface composition and the choice of adequate deposition conditions avoiding re-sputtering during the film growth. The increase in the constant phase element related to the double layer depending on the RE element deposited on the surface substrates indicates a high level of heterogeneity in the interfacial region, suggesting that more water is penetrating into the RE coating, which in turn reduced the polarization resistance. For both aluminum substrates, sputtered thinner cerium films were less effective in avoiding the corrosion activity in comparison with lanthanum coatings, which seem to interact better with the substrate surface during film formation.

Finally, the electrochemical results showed a reduction in the beneficial effect to delay pitting corrosion of aluminum alloys when ceria and lanthanum thin films are intercalated in comparison with La films, however, La/Ce and Ce/La oxide bilayered coatings displayed better results in terms of corrosion resistance than those observed for Ce coatings. In general, a compact morphology (smooth) is necessary to reduce the aggressive ion diffusion before reaching the metal substrate.

Based on the morphological observations, it is evident that the power required for the deposition of the second layer provokes erosion of the first layer and a less dense film with pores. Although, specific studies are required to determine the main factors that affect the morphology during bilayer formation.

## 4. Conclusions

La, Ce, La/Ce and Ce/La oxide coatings were deposited on aluminum substrates (AA7075 and AA6061) using RF magnetron sputtering under different deposition conditions. Stoichiometric La_2_O_3_ coatings with a hexagonal structure are mainly transformed into La(OH)_3_ when exposed to the atmosphere. Small peaks of lanthanum carbonates with monoclinic structure were also detected. The reactivity and crystallinity of La films to form La(OH)_3_/La_2_O_2_CO_3_ can be fitted by adjusting the sputtering parameters.

Nanocrystalline cerium oxide films with orientation in the (111) and (311) planes were detected by GIXRD indicating that Ce films produced at 80 W were crystalline whereas at 60 W, only at high temperature, oriented films were observed. XRD peaks corresponding to the crystalline hexagonal Ce_2_O_3_ phase were missing, but the XPS measurements confirmed the formation of both Ce^4+^ and Ce^3+^ species during the sputtering process. Ce^3+^ species can be formed as Ce_2_O_3_ in an amorphous phase or around oxygen vacancies of CeO_2_ compounds. The Ce^4+^/Ce^3+^ ratio in the films varied from 0.97 to 2.84 which corroborated the predominance of Ce^4+^ compounds.

AFM and SEM observations evidenced a typical Stranski–Krastanov type growth that occurs from a rapid deposition of irregular particles to form islands followed by thickening until the entire substrate is covered. This growth mechanism remained unchanged during the deposition of La/Ce and Ce/La bilayered coatings.

The evaluated parameters could be modulated to obtain slightly rough surfaces with more compact morphology, however, the substrate composition in combination with the applied power resulted in determinant factors to inhibit the kinetics of O_2_ reduction/H_2_ evolution reactions. AA6061 substrates increased the growth rate of the RE layers and favored the deposition of energetic atoms as stable growth grains in comparison with AA7075 samples.

The deposition of lanthanum coatings on both metallic substrates favored the well-known, self-healing properties in aggressive media. La coatings synthesized at *P_60_T_80_t_60_* provided an enhancement of up to three orders of magnitude in the barrier properties in comparison with uncoated specimens and displayed better electrochemical behavior that Ce coatings at similar deposition parameters. The formation of insoluble and stable lanthanum hydroxides propitiated an increase in the protective properties of the double layer coating.

The EIS results showed a reduction in the beneficial effect when cerium and lanthanum thin films are intercalated delaying pitting corrosion of aluminum alloys; unfortunately, RE bilayered coatings obtained by RF magnetron sputtering did not improve significantly the inhibition of the corrosion process of aluminum in a chloride medium.

Finally, these results show that the efficiency of RE-sputtered coatings to inhibit the corrosion process of aluminum in chloride media is strongly influenced by the deposition parameters, substrate composition, and in the case of bilayered coatings, the way in which they are deposited.

## Figures and Tables

**Table 1:** Crystallite size, thickness and Ra of sputtered RE coatings at different experimental conditions.

**Sample**	**P (W)**	**Time (min)**	**Deposition Temperature (°C)**							
			**80**				**200**			
			**Crystallite Size**		**Thickness**	**R_a_**	**Crystallite Size**		**Thickness**	**R_a_**
			**(nm)**		**(nm)**	**(nm)**	**(nm)**		**(nm)**	**(nm)**
AA6061/Ce	60	25	-		19.3	176.0	7.0		12.7	70.8
		40	-		30.8	88.2	1.1		22.9	95.4
		60	-		32.4	65.1	5.5		40.1	59.6
	80	25	-		44.6	289.0	-		42.2	131.6
		40	-		52.8	258.0	-		46.1	44.2
		60	-		149.1	127.9	-		158.1	112.2
**Sample**	**P (W)**	**Time (min)**	**Deposition Temperature (°C)**							
			**80**			**200**				
			**Crystallite Size**		**Thickness**	**R_a_**	**Crystallite Size**		**Thickness**	**R_a_**
			**(nm)**		**(nm)**	**(nm)**	**(nm)**		**(nm)**	**(nm)**
			**La_2_O_3_**	**La(OH)_3_**			**La_2_O_3_**	**La(OH)_3_**		
AA6061/La	60	25	9.2	12.8	269.5	45.0	6.3	6.5	354.3	36.1
		40	9.1	9.8	337.5	74.2	2.6	2.6	310.1	98.5
		60	5.3	2.8	390.2	66.0	2.0	2.0	349.1	108.0
	80	25	6.2	7.0	528.5	102.0	3.5	3.3	499.0	166.0
		40	3.2	4.1	545.2	186.0	2.8	2.8	670.8	108.4
		60	1.5	2.1	750.6	141.0	0.9	1.0	835.5	111.0
***T* = 80 °C, *t* = 60 min**										
**Bilayered Coatings**		**Thickness (nm)**			**R_a_ (nm)**			**Maximum Peak to Valley Height (nm)**		
AA6061/(Ce (*P_80_T_80_t_60_*)/La(*P_60_T_80_t_60_*)		268.6			67.1			700.0		
AA7075/(Ce (*P_80_T_80_t_60_*)/La(*P_60_T_80_t_60_*)		182.1			71.4			400.0		
AA6061/(La(*P_60_T_80_t_60_*)/(Ce (*P_80_T_80_t_60_*)		284.3			41.7			390.0		
AA7075/La(*P_60_T_80_t_60_*)/(Ce (*P_80_T_80_t_60_*)		193.1			27.7			204.0		

**Table 2:** Determination of Ce^4+^/Ce^3+^ and La oxide/hydroxide ratios from XPS measurements.

Sample	Ce^4+^/Ce^3+^ Ratio	La Oxide/Hydroxide Ratio
*P*_60_ *T*_80_ *t*_25_	1.69	0.72
*P*_60_ *T*_80_ *t*_40_	2.83	0.78
*P_60_T_80_t_60_*	1.54	0.62
*P*_80_ *T*_80_ *t*_25_	1.44	0.77
*P*_80_ *T*_80_ *t*_40_	2.26	0.69
*P*_80_ *T*_80_ *t*_60_	1.53	0.76
*P*_60_ *T*_200_ *t*_25_	0.97	0.65
*P*_60_ *T*_200_ *t*_40_	2.84	0.71
*P*_60_ *T*_200_ *t*_60_	1.24	0.78
*P*_80_ *T*_200_ *t*_25_	--	0.81
*P*_80_ *T*_200_ *t*_40_	2.04	0.66
*P*_80_ *T*_200_ *t*_60_	1.60	0.63

**Table 3:** *E*_corr_, *i*_corr_ and corrosion rate calculated from the potentiodynamic curves.

Sample	*β_a_*	*β*_c_	*i*_corr_	*E*_corr_	IE
	(mV/dec)	(mV/dec)	(nA cm^−2^)	(mV_SCE_)	(%)
AA7075					
Substrate	41.3	--	2297.1	−806	--
Ce (*P*_80_*T*_80_*t*_60_)	60.7	--	1440.6	−742	37.28
La (*P*_60_*T*_80_*t*_60_)	90.6	--	78.4	−731	96.6
Ce/La(*P*_80_*T*_80_*t*_60_)/(*P*_60_*T*_80_*t*_60_)	92.1	--	949.8	−746	58.6
La/Ce (*P*_60_*T*_80_*t*_60_)/(*P*_80_*T*_80_*t*_60_)	92.8	360.1	566.6	−751	75.3
AA6061					
Substrate	93.9	--	963.1	−863	--
Ce (*P*_80_*T*_80_*t*_60_)	105.0	--	524.5	−694	45.7
La (*P*_60_*T*_80_*t*_60_)	39.3	408.5	17.7	−932-	98.2
Ce/La (*P*_80_*T*_80_*t*_60_)/(*P*_60_*T*_80_*t*_60_)	93.9	--	195.4	−928	79.7
La/Ce (*P*_60_*T*_80_*t*_60_)/(*P*_80_*T*_80_*t*_60_)	35.1|	--	99.1	−719	89.7

**Table 4:** Fitting analysis of EIS spectra with their corresponding equivalent circuits.

Sample	*R*_s_ (Ω cm^2^)	*R*_film_ (Ω cm^2^)	*R*_ox_ (Ω cm^2^)	*R*_ct_ (Ω cm^2^)	*CPE*_film_ Ω^−1^ cm^−2^s^n^	*n*_film_	*CPE*_ox_ Ω^−1^ cm^−2^s^n^	*n*_ox_	*CPE*_dl_ Ω^−1^ cm^−2^s^n^	*n*_dl_	*χ* ^2^	Equivalent Circuit
AA7075												
Bare aluminum	16.1	--	38.3	7.3 × 10^3^	--	--	87 × 10^−6^	0.87	1.9 × 10^−6^	0.86	1.01 × 10^−3^	(a)
La (*P*_60_*T*_80_*t*_60_)	16.7	1.3 × 10^6^	514.4	1.8 × 10^6^	29.8 × 10^−9^	0.96	57 × 10^−6^	0.75	115.1 × 10^−9^	0.72	1.36 × 10^−3^	
Ce (*P*_80_*T*_80_*t*_60_)	14.8	8.9 × 10^3^	115.1	12.9 × 10^3^	3.0 × 10^−6^	0.85	96 × 10^−6^	0.84	0.34 × 10^−8^	0.67	0.56 × 10^−3^	(b)
Ce/La(*P*_80_*T*_80_*t*_60_)/(*P*_60_*T*_80_*t*_60_)	15.9	9.8 × 10^3^	756.3	16.3 × 10^3^	0.13 × 10^−6^	0.66	0.15 × 10^−6^	0.84	2.1 × 10^−6^	0.67	1.64 × 10^−3^	
La/Ce(*P*_60_*T*_80_*t*_60_)/(*P*_80_*T*_80_*t*_60_)	14.0	7.7 × 10^4^	598	6.6 × 10^4^	61.3 × 10^−6^	0.88	68.4 × 10^−6^	0.77	61.0 × 10^−7^	0.89	0.67 × 10^−3^	
AA6061												
Bare aluminum	15.1	--	630.0	2.0×10^3^	--	--	9.6 × 10^−5^	0.88	4.3 × 10^−6^	0.85	7.9 × 10^−3^	(a)
La (*P*_60_*T*_80_*t*_60_)	15.2	20.8 × 10^3^	132.5	2.3×10^6^	1.3 × 10^−6^	0.86	12.2 × 10^−9^	0.86	6.4 × 10^−6^	0.86	3.37 × 10^−3^	
Ce (*P*_80_*T*_80_*t*_60_)	14.3	5.7 × 10^3^	282.2	2.2×10^5^	2.6 × 10^−6^	0.87	170.1 × 10^−9^	0.70	13.2 × 10^−7^	0.87	2.7 × 10^−3^	(b)
Ce/La (*P*_80_*T*_80_*t*_60_)/(*P*_60_*T*_80_*t*_60_)	15.3	8.0 × 10^3^	383.5	5.2×10^5^	6.6 × 10^−6^	0.89	2.7 × 10^−6^	0.78	21.0 × 10^−6^	0.79	1.39 × 10^−3^	
La/Ce (*P*_60_*T*_80_*t*_60_)/(*P*_80_*T*_80_*t*_60_)	15.7	13.2 × 10^3^	1120.1	3.5×10^5^	40.8 × 10^−6^	0.89	2.1 × 10^−6^	0.78	5.8 × 10^−6^	0.83	1.07 × 10^−3^	

## Supplementary Materials

The following are available online at http://www.mdpi.com/1996-1944/11/7/1114/s1, Figure S1: Magnification of XRD patterns of lanthanum coatings on glass substrates under different experimental conditions, Figure S2: Low XPS resolution of selected Ce coatings on aluminum substrates, Figure S3: High XPS resolution and deconvolution of selected Ce coatings on aluminum substrates. Figure S4: AFM images of sputtered RE films deposited onto AA6061 aluminum alloys using the evaluated deposition conditions.
