# Supplementary material for: Fabrication of Sputtered Ce/La, La/Ce Oxide Bilayers on AA6061 and AA7075 Aluminum Alloys for the Development of Corrosion Protective Coatings
Source: Materials (Basel). 2018 Jun 29;11(7):1114. doi: 10.3390/ma11071114 (PMC6073953; doi:10.3390/ma11071114)
Supplement: Supplementary file 1 [file materials-11-01114-s001.pdf]

Fabrication of Sputtered Ce/La, La/Ce Oxide Bilayers on AA6061 and AA7075 Aluminum Alloys for the Development of Corrosion Protective Coatings

S.B. Brachetti-Sibaja [1,2], M. A. Domínguez-Crespo [1,*], A.M. Torres-Huerta [1], S. E. Rodil[3], A. B.López-Oyama [4], D.S. García-Zaleta [5] and E. Onofre-Bustamante [1]

Supplementary Materials:

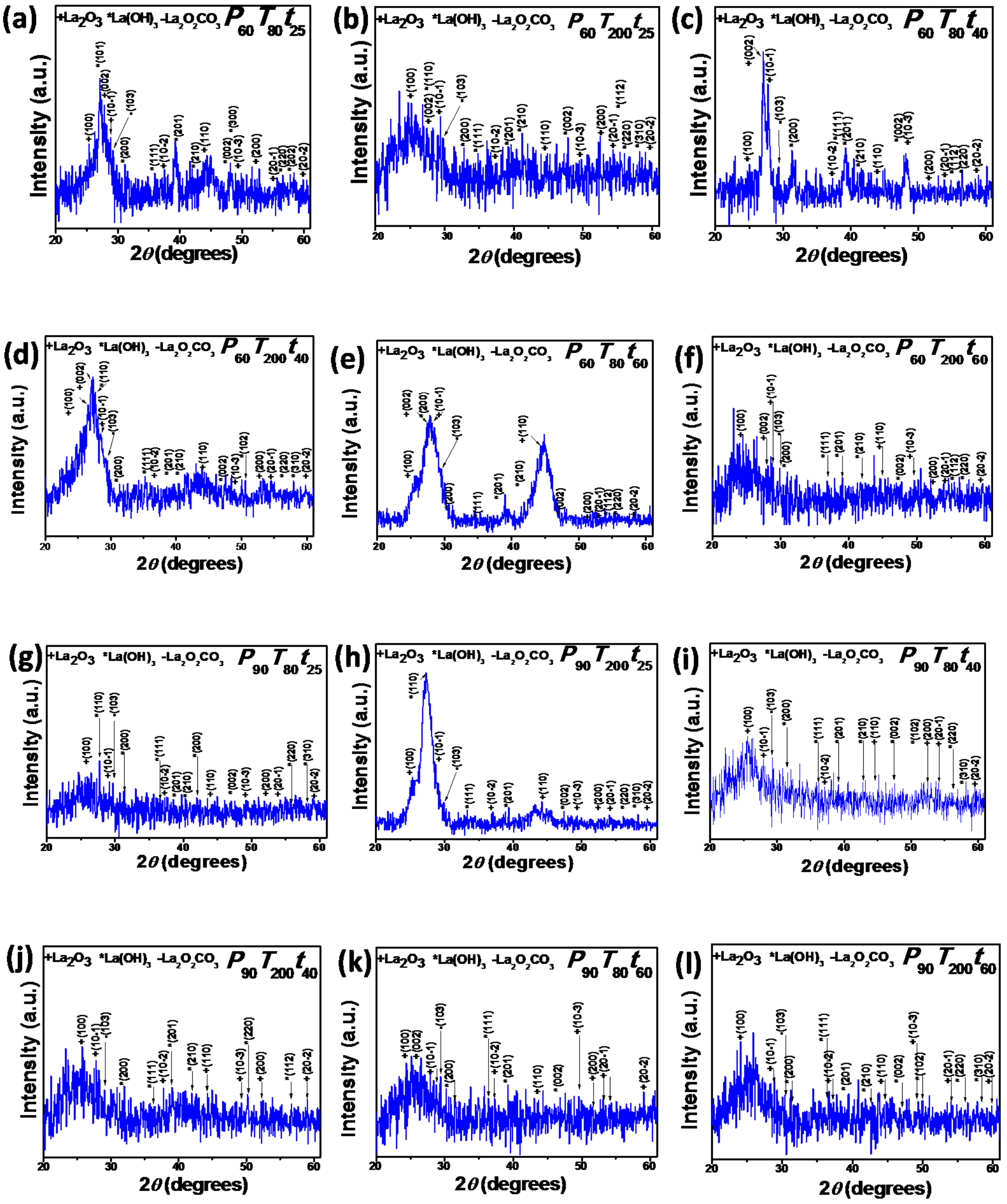

Figure S1. Magnification of XRD patterns of lanthanum coatings on glass substrates under different experimental conditions.

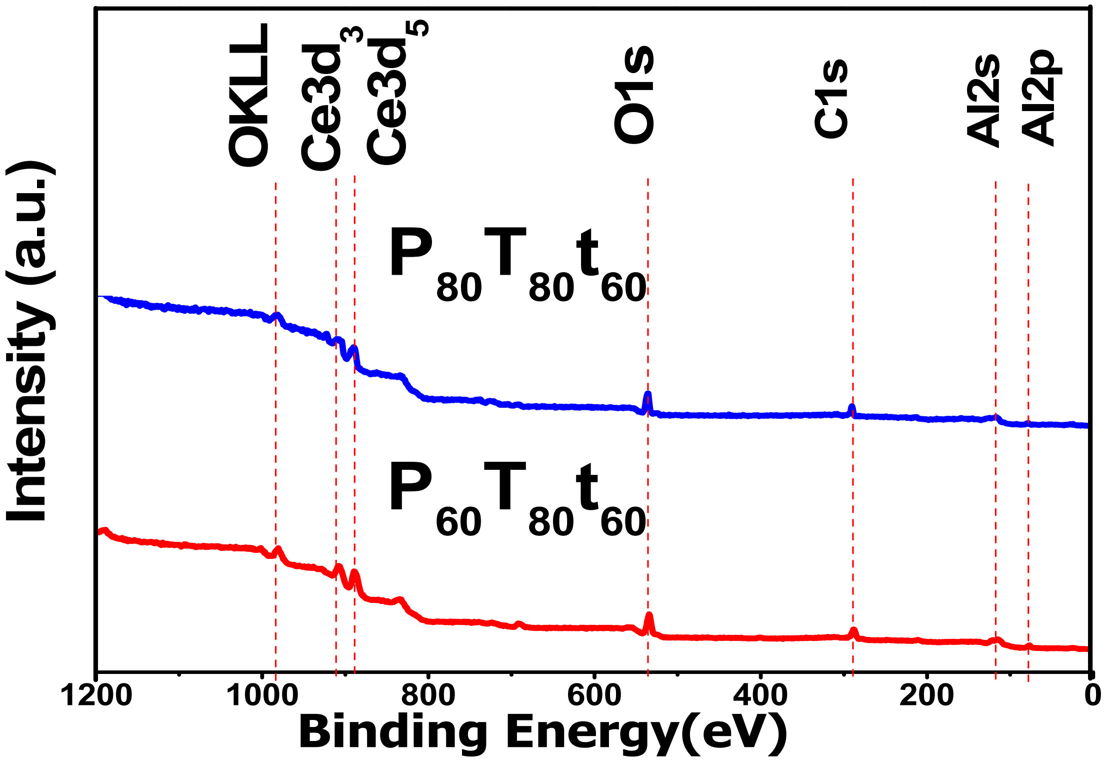

Figure S2. Low XPS resolution of selected Ce coatings on aluminum substrates.

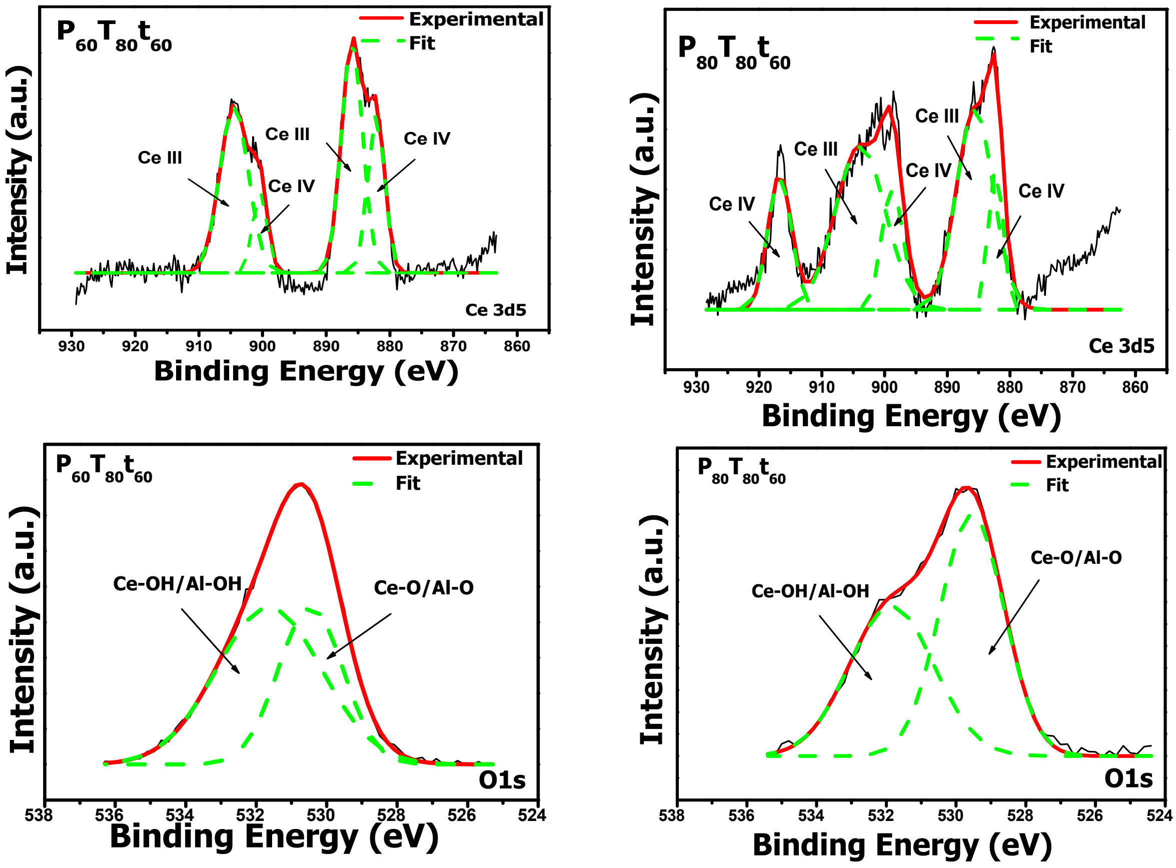

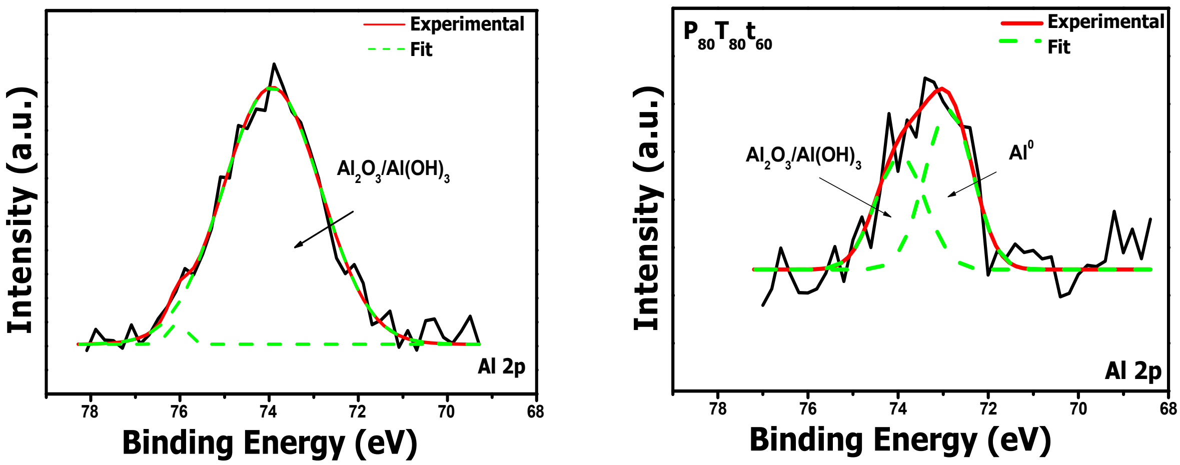

Figure S3. High XPS resolution and deconvolution of selected Ce coatings on aluminum substrates.

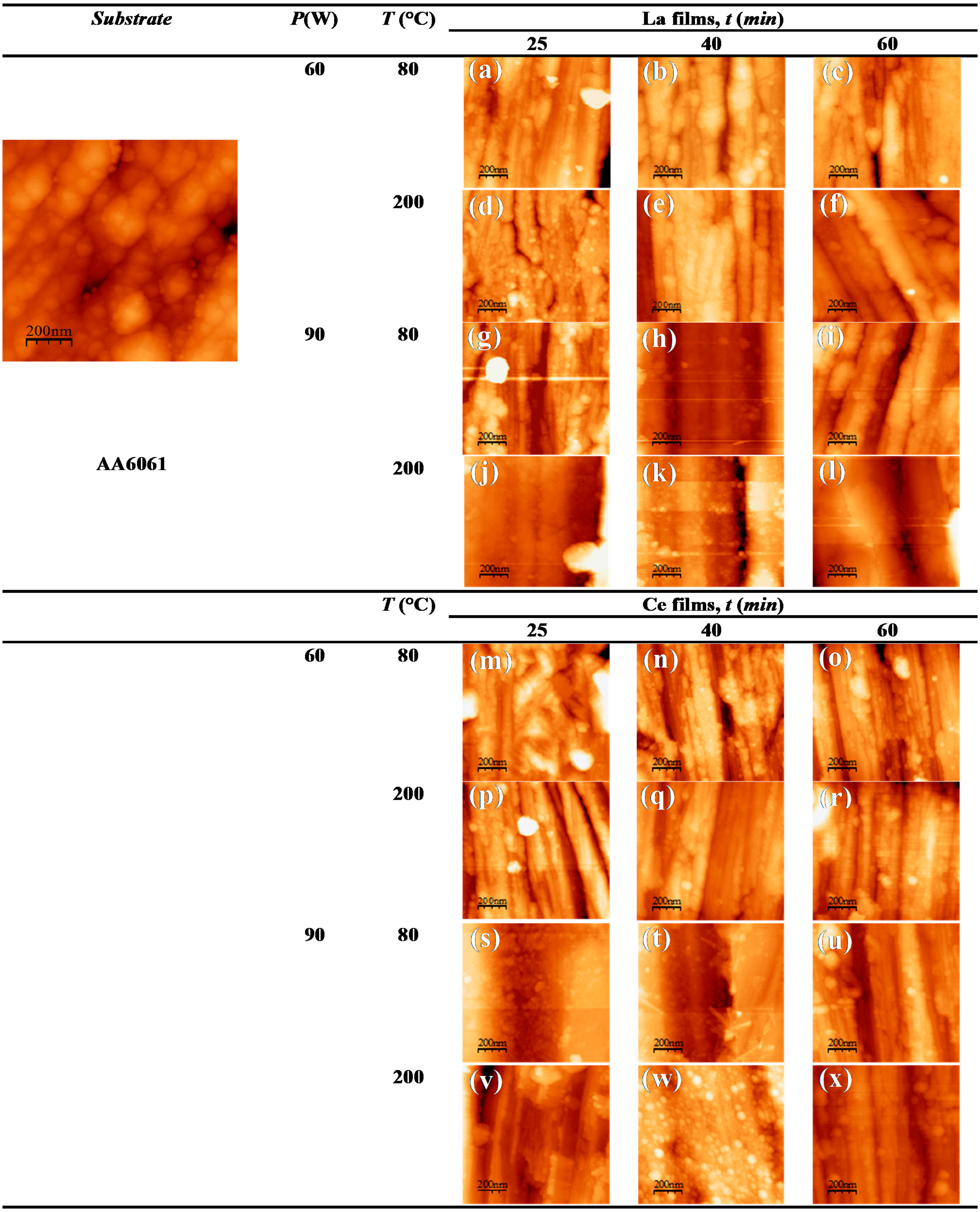

Figure S4. AFM images of sputtered RE films deposited onto AA6061 aluminum alloys using the evaluated deposition conditions.
